# Thyroid hormone inhibits growth of hepatoma cells through induction of miR-214

**DOI:** 10.1038/s41598-017-14864-1

**Published:** 2017-11-01

**Authors:** Po-Shuan Huang, Yang-Hsiang Lin, Hsiang-Cheng Chi, Pei-Yu Chen, Ya-Hui Huang, Chau-Ting Yeh, Chia-Siu Wang, Kwang-Huei Lin

**Affiliations:** 1 Department of Biochemistry, College of Medicine, Chang-Gung University, 333 Taoyuan, Taiwan; 2 Radiation Biology Research Center, Institute for Radiological Research, Chang Gung University/Chang Gung Memorial Hospital, 333, Linkou, Taoyuan Taiwan; 3 Liver Research Center, Chang Gung Memorial Hospital, 333, Linko, Taoyuan Taiwan; 4 Department of General Surgery, Chang Gung Memorial Hospital, Chiayi, 613 Taiwan; 5 Research Center for Chinese Herbal Medicine, College of Human Ecology, Chang Gung University of Science and Technology, 333, Taoyuan, Taiwan

## Abstract

Thyroid hormone (TH) plays a role in regulating the metabolic rate, heart functions, muscle control and maintenance of bones. 3,3′5-tri-iodo-L-thyronine (T_3_) displays high affinity to nuclear thyroid hormone receptors (TRs), which mediate most TH actions. Recent studies have shown hypothyroidism in patients with an increased risk of hepatocellular carcinoma (HCC). MicroRNAs (miRNAs), a class of non-protein-coding RNA, are suggested to control tumor growth by interacting with target genes. However, the clinical significance of T_3_/TR-regulated miRNAs in tumors has yet to be established. In the current study, miRNA expression profile screening was performed using SYBR Green-Based qRT-PCR array in TR-overexpressing HepG2 cells. miR-214-3p, which is expressed at low levels in HCC, was stimulated upon T_3_ application. The 3′UTR luciferase reporter assay confirmed that the proto-oncogene serine/threonine-protein kinase, PIM-1, is a miR-214-3p target. PIM-1 was decreased upon treatment with miR-214-3p or T_3_ stimulation. PIM-1 was highly expressed in HCC, and the effect of PIM-1 on cell proliferation might be mediated by the inhibition of p21. Furthermore, the T_3_-induced suppression of cell proliferation was partially rescued upon miR-214-3p knockdown. Our data demonstrate that T_3_ induces miR-214-3p expression and suppresses cell proliferation through PIM-1, thus contributing to the inhibition of HCC tumor formation.

## Introduction

Thyroid hormone, 3,3′,5-tri-iodo-L-thyronine (T_3_) is a potent mediator of several physiological processes, including embryonic development, cellular differentiation, metabolism and cell growth. Thyroid hormone receptors (TR) are a nuclear receptor superfamily that exert biological functions through transcriptional regulation. Human TRs are encoded by two separate isoform genes, THRA and THRB, which are located on human chromosomes 17 and 3, respectively, and are generated by alternative splicing and different promoter choices. The two genes yield four protein products, designated TRα1, TRα2, TRβ1, and TRβ2^1^. T_3_ and the TRs regulate gene transcription by binding the thyroid hormone response elements (TREs), which are located in the upstream promoter regions of the target genes. Mutational analyses of rat growth hormone TREs from other T_3_-responsive genes have led to the identification of a putative consensus hexamer half-site sequence, (G/A)GGT(C/G)A^2^. In particular, TRs bind TREs, in which half-sites are arranged as palindromes (TRE_pal_), direct repeats (DRs), and inverted palindromes (IPs)^2^. In positively regulated genes, TRs recruit co-repressors to suppress gene transcription in the absence of T_3_ but release co-repressors and recruit co-activators that stimulate gene transcription in the presence of T_3_^3^.

Several controversial studies have been published regarding the relationship between thyroid hormone levels and human cancer^4^. Data from animal models and epidemiologic studies indicate an association between higher thyroid hormone levels and the prevention of liver diseases^5,6^. Dickkopf (DKK) 4, a secreted protein that antagonizes the canonical Wnt signaling pathway, is induced by T_3_/TR at both the mRNA and protein levels in HCC cell lines^7^. T_3_/TR signaling suppresses cell proliferation by upregulating endoglin, thereby affecting p21 stability^8^. The collective findings suggest that the aberrant expression of T_3_/TR contributes to liver cancer^9^. However, TRs are also implicated in association with MAPK for glioma cells and β-catenin for intestinal epithelial cells^10,11^. Interestingly, T_3_ is reported to enhance proliferation in glioma and breast cancer cells^12^, suggesting a dual role of TRs during tumorigenesis in different cancer and disease types.

MicroRNA (miRNAs) are small non-coding RNAs that function in RNA silencing and the post-transcriptional regulation of gene expression^13^. MiRNAs bind the 3′ untranslated region (3′UTR) of the mRNA of target genes, which leads to translational repression or mRNA degradation^13,14^. The dysregulation of miRNAs is proposed to be associated with cancer formation. Recent studies have reported that circulating miRNAs serve as potential clinical biomarkers^15^. The functions of miRNAs in tumorigenesis have been investigated in various cancers, including hepatocellular carcinoma (HCC). In the current study, miR-214, which is expressed at low levels in human HCC and is upregulated by T_3_/TR, was selected for further analysis. The miR-214 and miR-199 clusters are located on the opposite strands of the Dynamin3 gene (DNM3)^16^. miR-214 plays an important role in cancer progression and disease severity. Moreover, miR-214 is overexpressed in ovarian and oral mucosal cancers and in malignant melanomas^17–19^. miR-214 inhibits angiogenesis via the suppression of the target gene-HDGF in HCC and is associated with tumor progression and clinical outcome^20^. The expression of miR-214 is significantly associated with α-fetoprotein (AFP), which is commonly used as a marker for surveillance in high-risk HCC cases through its presence in serum and other body fluids^20,21^.

PIM-1 is a serine/threonine protein kinase proto-oncogene^22^. The expression of PIM-1 is induced by a variety of growth factors, cytokines, mitogens and hormones. PIM-1 regulates anti-apoptotic activity, cell cycle, and migration through the JAK/STAT pathway^23,24^. Data obtained from clinical studies confirm high expression levels of PIM-1 and support its utility as a prognostic biomarker in prostate cancer, oral cancer, colon cancer, head-and-neck squamous cell carcinoma, and gastric cancer^25–29^. However, the mechanisms underlying PIM-1 signaling in HCC remain to be established^30–34^.

## Results

### miR-214 is upregulated by T_3_

**Figure 1:**
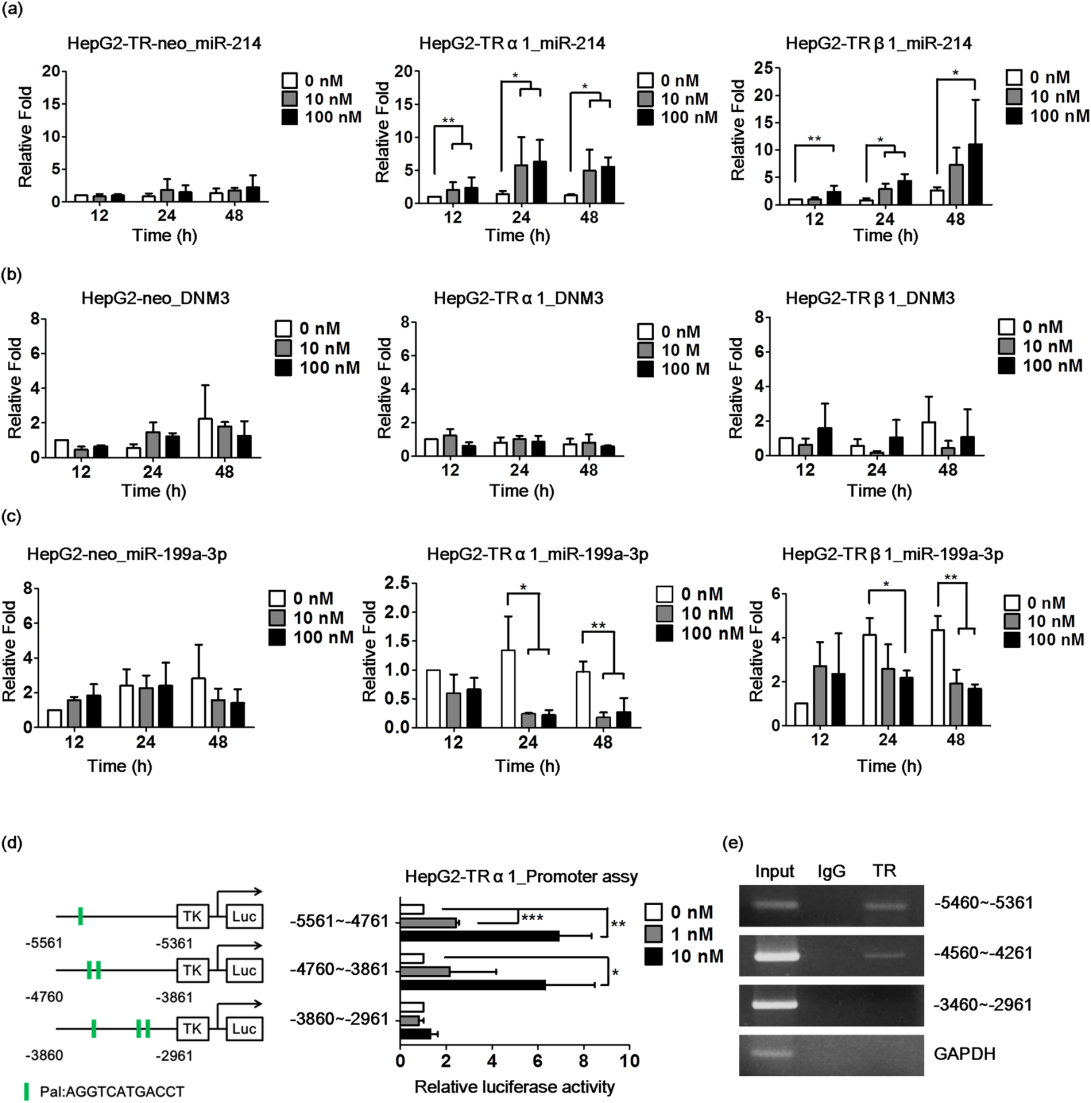
miR-214 is upregulated by T_3_. HepG2-TR cell lines were treated with 0-100 nM T_3_ for 12-48 hrs. (**a**) miR-214, (**b**) DNM3 and (**c**) miR-199a-3p were measured by qRT-PCR. U6 was used as a loading control. (**d**) HepG2-TRα1 cells were transfected with miR-214 reporter plasmids. The cells were incubated for 24 hrs with thyroid hormone (0–10 nM). (**e**) A ChIP assay was used to confirm that TR was directly bound to the miR-214 promoter region. GAPDH was the negative control. The data are presented as the means ± s.d. of three independent experiments (*p < 0.05; **p < 0.01; ***p < 0.001 vs. 12 hrs, 0 nM).

miR-214 was selected for study to demonstrate the potential suppressive role of the TR in view of its downregulation in HCC and positive regulation by T_3_/TR. To further ascertain whether T_3_/TRs upregulates miR-214 expression in HepG2-TR cells, experiments were performed at various time periods or T_3_ doses. Notably, the miR-214 level was significantly increased in HepG2-TRα1 and HepG2-TRβ1 cell lines in a time- and dose-dependent manner. In contrast, miR-214 was only marginally upregulated by T_3_/TR in HepG2-neo cells devoid of TR expression (Fig. 1a). Further, the host gene, DNM3, was not affected (Fig. 1b), but miR-199a was downregulated by T_3_ (Fig. 1c). However, two TRE regions (−5460~−5361 and −4560~−4261) in the miR-214 upstream promoter were validated and confirmed by the luciferase reporter and chromatin immunoprecipitation (ChIP) assays (Fig. 1d,e).

### miR-214 inhibits the proliferation of HCC cell lines

**Figure 2:**
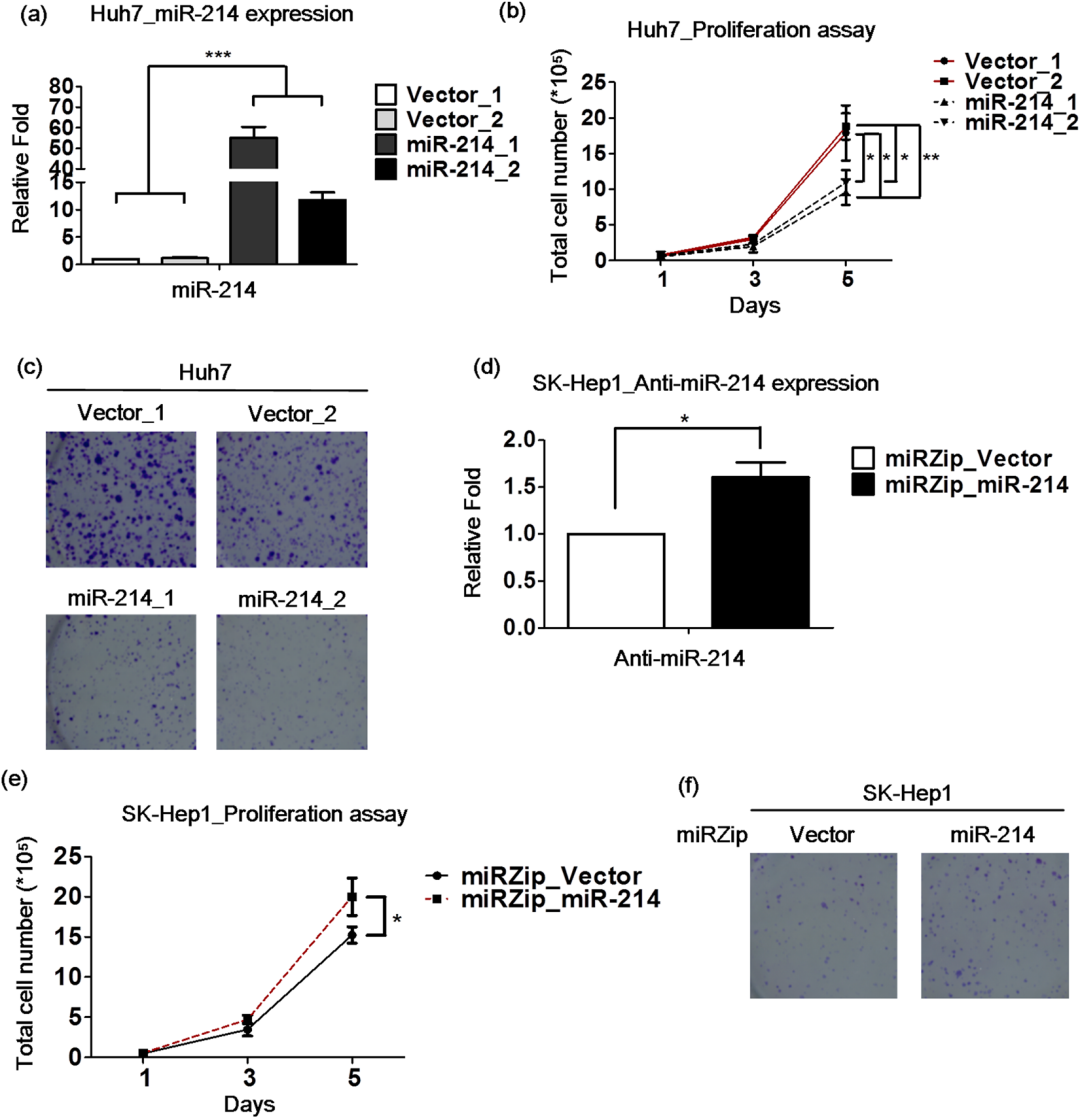
miR-214 inhibits the proliferation of HCC cell lines. Huh7 cell lines stably expressing miR-214 were established by transfection with the miR-214 plasmid. (**a**) The expression levels of miR-214 were measured by qRT-PCR and the vector pcDNA6.2 was used as a control, respectively. (**b**) The Huh7 hepatoma cell line proliferation capacity was measured by the total cell numbers. (**c**) Colony assay in the Huh7 cells. (**d**) The stable knockdown of miR-214 in the SK-Hep1 cell lines was established by infection with the lentivirus miRZip-miR-214 (Anti-miR-214). The expression levels of anti-miR-214 were measured by qRT-PCR. U6 was used as a loading control. (**e**) The SK-Hep1 hepatoma cell lines proliferation capacity was measured. (**f**) The colony assay in SK-Hep1. The data are presented as the means ± s.d. of three independent experiments (*p < 0.05; **p < 0.01; ***p < 0.001 vs. control vector).

The thyroid hormone suppresses HCC cell proliferation both *in vitro* and *in vivo* ^8^. To determine whether miR-214 is involved in the T_3_/TR-mediated reduction in cell growth, its effect on proliferation was examined. HCC cell lines expressing low (Huh7) and high (SK-Hep1) levels of miR-214 were used for the experiments. Huh7 cells stably overexpressing miR-214 were established (Fig. 2a). Notably, the overexpression of miR-214 in Huh7 cell lines suppressed proliferation and colony formation (Figs 2b,c and S1a). Conversely, SK-Hep1 cells with a stable knockdown of miR-214 were generated, and anti-sense miR-214 expression was detected by via qRT-PCR (Fig. 2d). The depletion of miR-214 led to an enhanced cell proliferation and colony formation in the SK-Hep1 cell line (Figs 2e,f and S1b). Our findings clearly indicate the tumor suppressor role of miR-214 in HCC cell lines.

### PIM-1 is the direct target of miR-214

**Figure 3:**
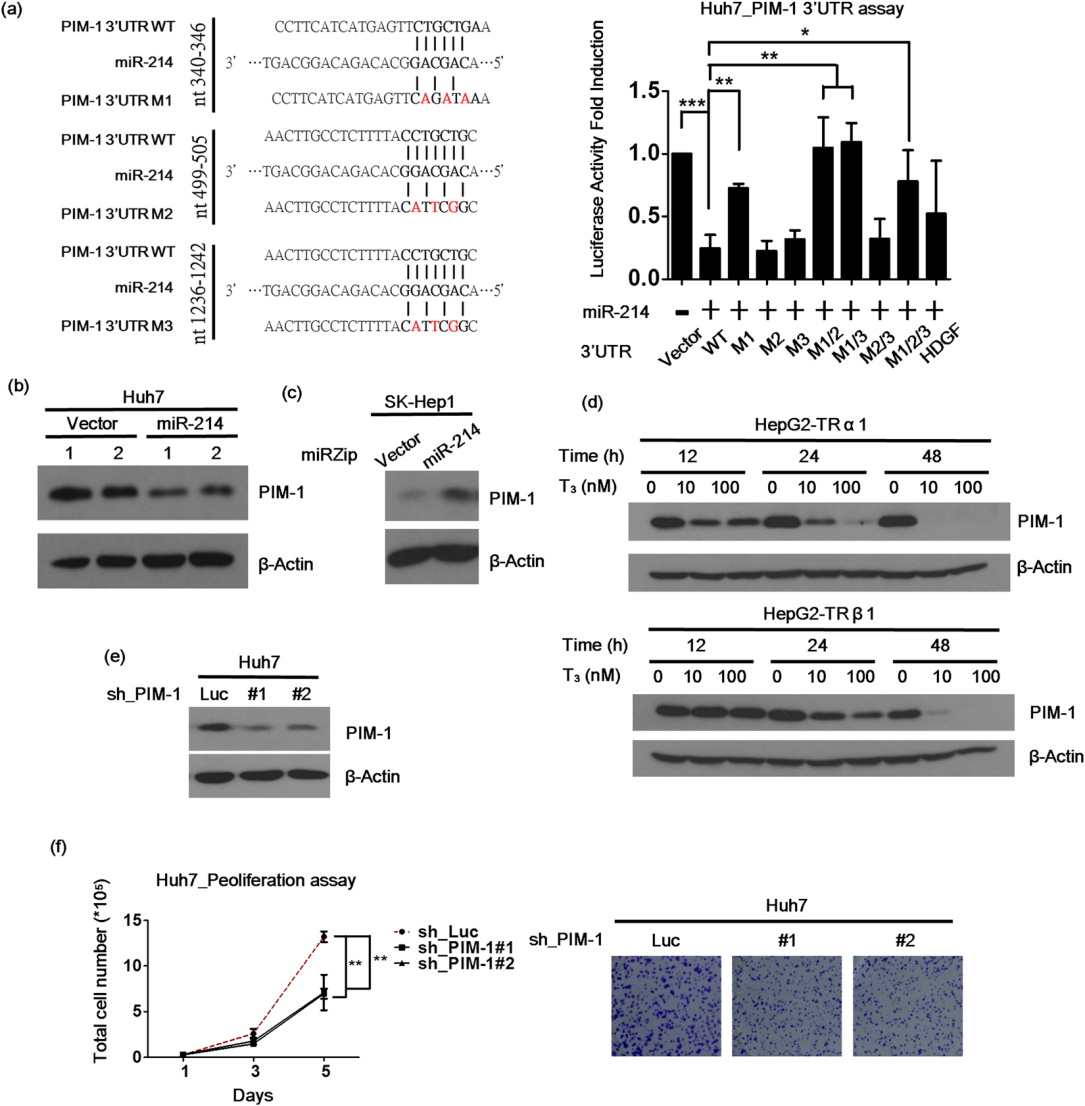
PIM-1 is the direct target of miR-214 and is regulated by T_3_ in HCC. (**a**) The wild-type (WT) and mutant (M1-M3) 3′UTRs of PIM-1 were cloned, and (left) the luciferase reporter assay was performed in the Huh7 cell line (right) that stably expressed miR-214 or the vector control. (**b**) PIM-1 protein levels in the Huh7 stable expressing and (**c**) SK-Hep1 stable knockdown miR-214 cells was measured by western blot. (**d**) The HepG2-TR cell lines were treated with 0-100 nM T_3_ for 12-48 hrs, and PIM-1 protein levels were measured by western blot. (**e**) The stable knockdown PIM-1 in the Huh7 cell lines was established by transfection with the shRNA PIM-1 plasmids. (**f**) The Huh7 hepatoma cell line proliferation capacity was measured by a proliferation assay or a colony formation assay. β-actin was used as a loading control. The data are presented as the means ± s.d. of three independent experiments (*p < 0.05; **p < 0.01; ***p < 0.001 vs. Vector), and the luciferase activity was normalized with beta-galactosidase.

To explore the specific effects of the T_3_/TR/miR-214 axis in hepatoma cells, the target genes of miR-214 were further investigated. The online database, TargetScan, was used to identify the potential target genes of miR-214, and the luciferase reporter assay performed to validate the regulated genes. Subsequently, the full-length wild-type 3′untranslated region (UTR, WT) and the mutant 3′UTR regions (M1, M2 and M3) of PIM-1 were cloned into a firefly luciferase reporter plasmid, as shown in Fig. 3a. The luciferase activity of the wild-type PIM-1 3′UTR was inhibited by miR-214. Additionally, the M1 mutant (including M1/2, M1/3 and M1/2/3) abolished this repression. However, the reporter activity of the M2 or M3 mutant was still repressed by miR-214. HDGF, a known miR-214 target gene, was used as the positive control (Fig. 3a). PIM-1 protein expression was further analyzed via western blot in miR-214 overexpressing and knockdown HCC cell lines. The expression of PIM-1 was clearly suppressed in miR-214-overexpressing cells (Figs 3b and S2a) and, conversely, was increased in SK-Hep1 cells that had been deleted of miR-214 (Figs 3c and S2b).

### PIM-1 is a proto-oncogene in HCC that is regulated by T_3_

PIM-1 was identified as a proto-oncogene in many cancers, including pancreatic, prostate, gastric and skin cancer and leukemia^25,35–38^, but its underlying mechanism of action in HCC remains to be established. The effects of T_3_/TR on PIM-1 expression were determined in HepG2-TR cells. Our data showed that PIM-1 expression is decreased in HepG2-TRα1 and HepG2-TRβ1 cell lines (Figs 3d and S3a). The stable depletion of PIM-1 was achieved and detected via western blot (Figs 3e and S3b,d). The knockdown of PIM-1 suppressed cell proliferation and colony formation (Figs 3f and S3c), supporting its role as a proto-oncogene in HCC.

### miR-214 suppresses cell proliferation by modulating the PIM-1 pathway

**Figure 4:**
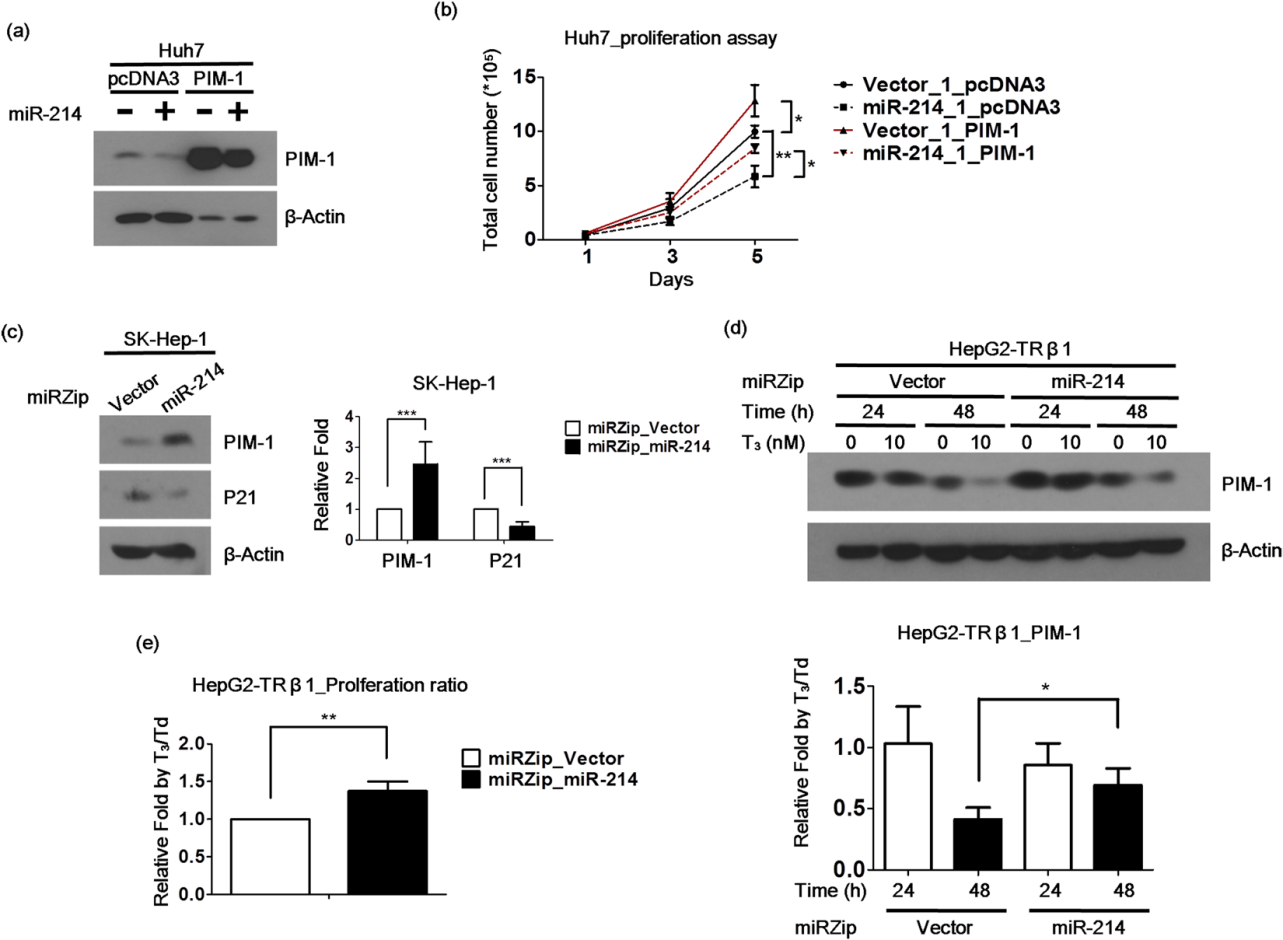
Thyroid hormone suppresses cell proliferation through the upregulation of miR-214 by modulating the PIM-1 pathway. (**a**) The re-expression of PIM-1 in the miR-214 stable Huh7 cell lines, and the PIM-1 protein levels were measured by western blot. (**b**) The proliferation capacity of the Huh7 hepatoma cell lines was measured by a proliferation assay or the colony formation assay with/without PIM-1 re-expression. (**c**) PIM-1 and p21 protein expression were measured in stable knockdown miR-214 SK-Hep1 or HepG2-TRα1 cell lines by western blot. (**d**) HepG2-TRβ1 depleted miR-214 cell lines were treated with 0-10 nM T_3_ for 24-48 hrs, and the PIM-1 protein levels were measured by western blot. β-actin was used as a loading control. The PIM-1 protein levels are shown by the relative fold of T_3_ depleted (Td)/T_3_. (**e**) HepG2-TRβ1 depletion of the miR-214 cell lines were treated with 0–10 nM T_3_, and the proliferation capacity was measured and is shown by the relative fold change of the Td/T_3_ ratio. The data are presented as the means ± s.d. of three independent experiments (*P < 0.05;**P < 0.01;***P < 0.001 vs. Vector).

To address the possible involvement of PIM-1 in the miR-214-mediated suppression of cell growth, PIM-1 was re-expressed in miR-214-overexpressing Huh7 cells (Figs 4a and S4a). Interestingly, the overexpression of miR-214 in Huh7 cells inhibited their cell proliferation capacity (Fig. 4b), which was rescued following the re-expression of PIM-1 (Fig. 4b). p21 is inhibited by PIM-1 in other cancer types, but its expression patterns in HCC are currently unclear. SK-Hep1 and HepG2-TRα1 cell lines with the miR-214 knockdown were established. Upon the suppression of miR-214, the expression of PIM-1 increased, and that of p21 decreased (Figs 4c and S4b). These findings suggest that miR-214 suppresses cell proliferation by modulating the PIM-1 pathway and that PIM-1 is a direct target gene of miR-214 in hepatoma cells.

### Thyroid hormone suppresses cell proliferation through the upregulation of miR-214

To ascertain whether T_3_ regulates PIM-1 through the induction of miR-214, miR-214 expression was depleted in HepG2-TRβ1 cell lines. The expression of the PIM-1 protein was suppressed by T_3_/TR, which was abolished upon miR-214 knockdown (Fig. 4d), indicating that T_3_ upregulates miR-214 and suppresses PIM-1 protein. Notably, the T_3_-induced inhibition of cell proliferation was restored upon the knockdown of miR-214 (Figs 4e and S4c). Based on the collective results, we propose that T_3_ suppresses cell proliferation through the miR-214-mediated repression of PIM-1.

### miR-214 reduces tumor formation *in vivo*

**Figure 5:**
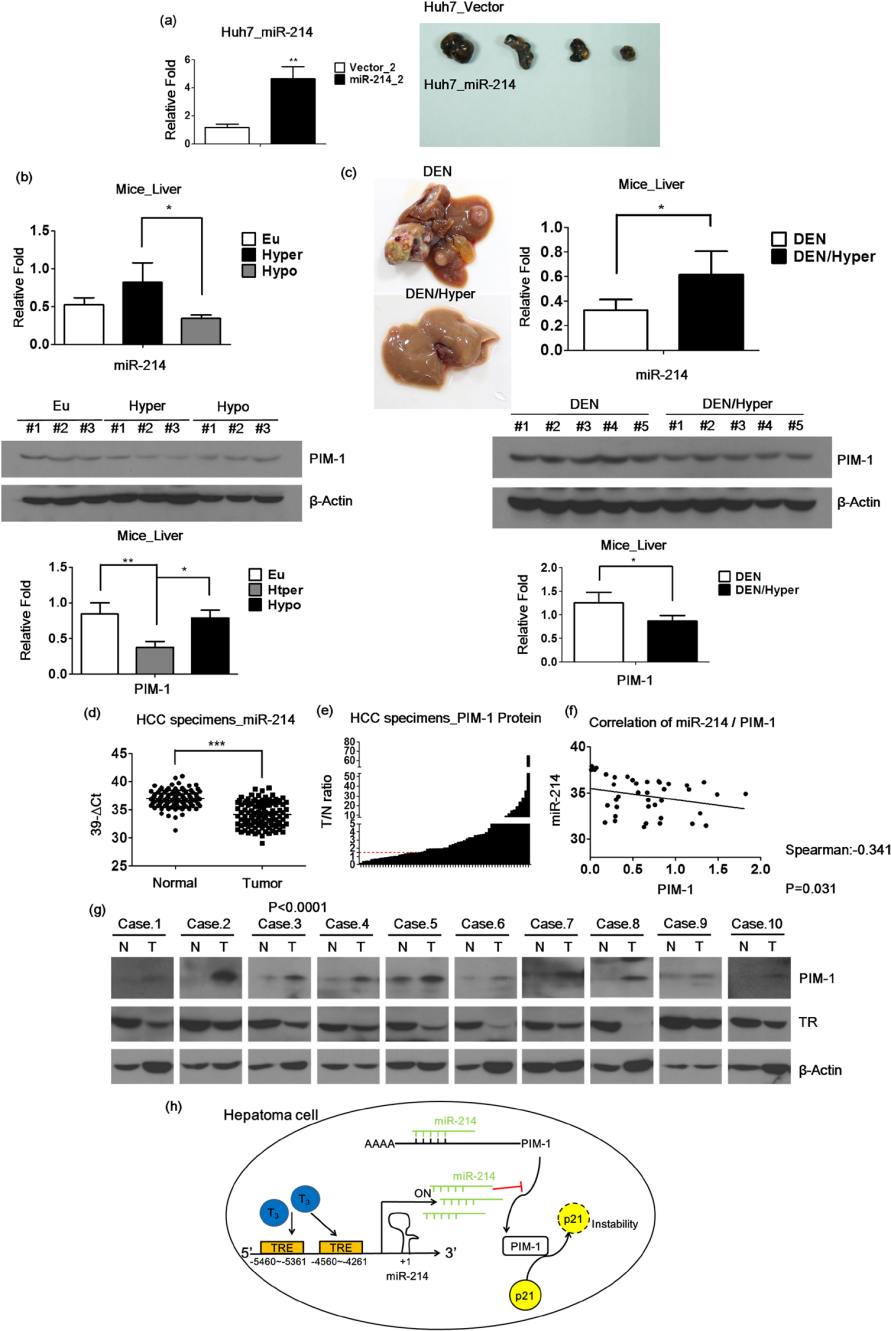
miR-214 reduces tumor formation *in vivo*. (**a**) Stable expressing miR-214 and the control (pcDNA6.2) Huh7 cell lines were subcutaneously injected into nude mice. (**b**) miR-214 expression was detected in the mice livers (n = 3) with Euthyroid (Eu), Hypothyroid (Hypo), and Hyperthyroid (Hyper) by qRT-PCR. (**c**) To detect miR-214 expression in the mice liver (n = 5) with DEN or DEN/Hyper, and the morphology of the livers, and the U6 was a loading control. The data are presented as the means ± s.d. (*p < 0.05; **p < 0.01; ***p < 0.001 vs. Hypo or DEN). (**d**) miR-214 expression levels were determined in 108 pairs of HCC specimens by qRT-PCR, and U6 was used as a loading control. The data are presented as 39-∆Ct (**e**) PIM-1 protein expression levels were determined in 61 pairs of HCC specimens by western blot, and β-actin was used as a loading control. The data are presented as the T/N ratio. (**f**) The correlation of miR-214 and PIM-1 in 40 pairs HCC specimens was analyzed by the Spearman’s rank correlation coefficient. (**g**) The PIM-1 and TR protein expressions are shown in 10 pairs of representative HCC specimens. (**h**) The cartoon illustrating the major observations reports that the thyroid hormone suppresses HCC cell proliferation by inducing miR-214 to repress PIM-1. (*p < 0.05;**p < 0.01; ***p < 0.001 vs. Vector or Normal).

To determine whether the *in vitro* effects of miR-214 can be replicated *in vivo*, xenograft mouse models were used. As expected, the overexpression of miR-214 in the Huh7 cell line led to the inhibition of tumor formation compared with the control group (Fig. 5a). Previously, to generate hyperthyroid HBx mice, T_3_ (2.5 mg/L) was added to the drinking water for 8 months. To induce hypothyroidism in mice, 0.02% methimazole plus 0.1% sodium perchlorate was added to the drinking water for 6 months^39^. Clearly, the data indicate that miR-214 was upregulated (Fig. 5b, upper panel) and that the target PIM-1 protein (Fig. 5b, lower panel) was downregulated by T_3_ *in vivo*. Additionally, in the mice treated with diethylnitrosamine (DEN) combined with a choline-deficient diet for weeks, multiple preneoplastic lesions were observed^40^. Notably, T_3_ plays a suppressor role to inhibit DEN-induced HCC, which is probably mediated by the upregulation of miR-214 (Fig. 5c, upper panel) to repress the target gene PIM-1 (Fig. 5c, lower panel) at the protein level.

The expression of miR-214 in clinical specimens was measured via qRT-PCR. The expression of miR-214 was lower (p < 0.0001, Fig. 5d), whereas that of PIM-1 was higher in the HCC tumors compared with the adjacent normal tissues (Fig. 5e); the inverse correlation is observed between PIM-1 and miR-214 (Fig. 5f). Moreover, PIM-1 expression was negatively correlated with TR (Fig. 5g). Our results further validate the tumor suppressor role of miR-214 *in vivo*. Additionally, the thyroid hormone suppresses HCC cell proliferation by inducing miR-214 to repress PIM-1(Fig. 5h).

## Discussion

In this study, we showed that miR-214, regulated by T_3_/TR, acts as a tumor suppressor in HCC cell lines. A previous study indicated that Twist1 interacts with the E-box of the miR-214/199a promoter region to stimulate the expression of the host gene DNM3os, encoding miR-214 and miR-199a in neural cells^41^. However, miR-199a expression was not upregulated by T_3_/TR. The differential regulation is due to TREs in the region between miR-199a and miR-214. Similarly, the miR-17-92 cluster containing six miRNAs is processed from the transcript of C13orf25 in lymphomas and solid tumors, but the expression is variable. These findings suggest that the processing or stability of miRNAs is differentially regulated^42,43^.

Several research groups have reported that the overexpression of miR-214 suppresses cell proliferation. miR-214 inhibits the expression of E2F2, CDK3 and CDK6 in HCC^44^. The reduced expression of miR-214, via the activation of the hepatoma-derived growth factor (HDGF), is observed in HCC^20,44^. miR-214 is additionally downregulated in colorectal cancer through promoter hypermethylation^45^. Lu *et al*.^46^ demonstrated that miR-214 downregulates UDP-N-acetyl-α-D-galactosamine: polypeptide N-acetylgalacto- saminyltransferase 7 (GALNT7) protein expression to suppress the invasive ability of esophageal squamous cell carcinoma. Furthermore, miR-214 mediates the downregulation of HMGA1 to inhibit growth and motility in human cervical and colorectal cancer cells^47^. The collective results are consistent with our finding that miR-214 plays a tumor suppressor role.

Conversely, the oncogenic role of miR-214 in osteosarcoma is reported^48^. miR-214 binds directly to the 3′UTR of LZTS1 mRNA to suppress its expression at both the transcriptional and translational levels, thus promoting osteosarcoma cell proliferation, invasion and tumor growth^49^. Alimirah and co-workers reported that a vitamin D receptor suppressed miR-214 but that the overexpression of miR-214 activated the hedgehog pathway to promote breast cancer progression. miR-214 acts as an oncogene in breast cancer to promote cell invasion through the downregulation of P53^50,51^. Moreover, miR-214 enhances the peritoneal metastasis of gastric cancer cells through the downregulation of PTEN expression^52^. However, miR-214 is downregulated by T_3_/TR in the mouse heart. Myocardial infarction (MI)-induced cardiac stress results in the activation of Dio3, leading to a reduced level of cardiac T_3_, which in turn, stimulates miR-214 expression^53^. The role of miR-214 in cardiac fibrosis, whereby miR-214 induces proliferation and collagen synthesis to contribute to cardiac injury, is not consistent with our finding in HCC^54^. We speculate that miRNA-214, which is regulated by T_3_, occurs in a tissue-specific manner. T_3_ induces cell proliferation to promote organ development in muscle, bone and heart^55–57^. However, according to our previous report, T_3_ represses cell proliferation in liver cancer cells^58^.

PIM-1 is the miR-214 target gene and is overexpressed in many cancer types, including prostate and breast cancers^22,59–62^. In our experiments, the PIM-1 protein levels were upregulated in the HCC specimens. The depletion of PIM-1 in HCC cell lines led to the suppression of the proliferation rate, suggesting that PIM-1 acts as a proto-oncogene in HCC. PIM-1 was further confirmed as a miR-214 target gene, consistent with an earlier study showing that is PIM-1 is a target gene of miR-214 in mesothelioma^63^. p21 is one of the downstream gene targets of PIM-1 in prostate, colon and lung carcinoma^64–66^. Here, the knockdown of miR-214 in HCC cell lines induced PIM-1 and the consequent inhibition of p21 expression. A previous study demonstrated that T_3_/TR suppresses cell proliferation by upregulating endoglin controlling p21 stability^8^. We propose that T_3_/TR regulates miR-214 expression to reduce PIM-1 expression and enhance p21 expression.

In conclusion, miR-214 was directly stimulated by T_3_ when its receptor bound to the upstream TRE in hepatoma cells. T_3_ suppressed proliferation by targeting miR-214 to downregulate PIM-1 in hepatoma cells. However, the reduced expression of TR led to the inhibition of miR-214 and, in turn, increased the expression of PIM-1 to promote tumorigenesis.

## Methods

### Cell culture and T_3_ treatment

Human hepatoma cell lines, HepG2, Huh7 and SK-Hep1, were routinely grown in DMEM supplemented with 10% fetal bovine serum. The HepG2 cell line used in this study was stably transfected with TRα (HepG2-TRα1) or TRβ (HepG2-TRβ1). HepG2-neo was used as the control cell line. The serum was depleted of T_3_ (Td) via a resin treatment. The cells were cultured at 37 °C in a humidified atmosphere of 95% air and 5% CO_2_.

### Establishment of miR-214 overexpressing and knockdown cells

The miR-214-expressing plasmid was cloned into the expression vector pcDNA6.2 followed by transfection into Huh7 cells using the TurboFect reagent (Thermo Fisher Scientific, Waltham, MA, USA). After 48 hrs of incubation, the cells were transferred to medium containing blasticidin for selection. After 2 weeks, the specific overexpression of miR-214 was confirmed via qRT-PCR. The lentivirus, miRZip-214, was purchased from System Biosciences (Mountain View, CA, USA). The plasmid and lentiviral package plasmids were cotransfected into Her-293T cells using the TurboFect reagent kit (Thermo Fisher Scientific) and produced viruses in the cells. After 24 hrs, the viral soup was collected for the infection of the HepG2-TR and SK-Hep1 cells. After 48 hrs of incubation, the cells were transferred into medium containing puromycin for selection. Specific anti-sense miR-214 was confirmed using qRT-PCR.

### Real-time polymerase chain reaction

Total RNA was purified using TRIzol reagent (Life Technologies Inc., Carlsbad, CA, USA) according to the supplier’s protocol, and cDNA was synthesized using a Superscript II kit (Life Technologies, Karlsruhe, Germany). The miRNA-specific stem-loop RT primers (miR-214-3p: CTCAACTGGTGTCGTGGAGTCGGCAATTC AGTTGAGCTGCCTGTCT;miR-199a-3p:CTCAACTGGTGTCGTGGAGTCGGCAATTCAGTTGAGTAACCAAT), dNTPs (10 nM), Superscript III reverse transcriptase and 1 µg total RNA were used for the microRNA RT reaction. All the reactions were conducted in an ABI PRISM 7500 sequencer (Applied Biosystems, Foster City, CA, USA).

### Promoter luciferase reporter analysis

Fragments of the miR-214 promoter were inserted into the PA3TK vector (Promega Corp, Madison, WI). To assess the influence of T_3_ on the transcriptional activity of the miR-214 promoter, HepG2-TRα1 cells were transfected with the miR-214 promoter using the TurboFect (Thermo Fisher Scientific) protocol. Twenty-four hours after the transfection, the cells were treated with Td/T_3_. Both the treated and untreated cells were incubated for a further 24 hrs and were lysed to measure the luciferase activity. Luciferase activity was assayed with a luminometer (LMax II 384; Molecular Devices, Sunnyvale, CA, USA).

### Chromatin immunoprecipitation (ChIP) assays

The ChIP assay was performed to detect the interactions between the TR and TREs on the miR-214 promoter regions, as previously described^67^.

### 3′UTR luciferase reporter analysis

Fragments of the PIM-1 3′UTR plasmid were inserted into the pMIR-REPORT vector (Applied Biosystems). Serial deletion mutants of the 3′UTR plasmid were amplified via PCR. To assess the interaction of miR-214 with the PIM-1 3′UTR, Huh7 overexpressing miR-214 cells were co-transfected with the PIM-1 3′UTR (WT and Mutant) and beta-galactosidase. At 24 hrs after the transfection, the cells were incubated for another 24 hrs and were lysed to measure the luciferase activity. Luciferase activity was assayed with a luminometer (LMax II 384; Molecular Devices, Sunnyvale, CA, USA).

### Cell proliferation and colony assays

The proliferation capacity of the hepatoma cell lines, under different the conditions, was assessed with the aid of the proliferation and colony assays. The final cell seed number was 3 × 10^4^ cells for the proliferation assay and 3 × 10^3^ cells for the colony assay. The cells were routinely grown in DMEM supplemented with 10% fetal bovine serum. For the proliferation assay, the cell cultures were treated with trypsin, and the detached cells were counted with a hemocytometer. The colonies were fixed and stained with crystal violet after two weeks.

### Mouse xenograft model

miR-214 overexpressing and control Huh7 cells (3 × 10^6^ cells/200 μl) were injected subcutaneously into the flanks of immunodeficient nude mice. All the procedures were carried out in accordance with the Guide for the Care and Use of Laboratory Animals issued by the Institutional Animal Care and Use Committee of Chang Gung University and the National Institutes of Health of United States. All methods were approved by the Chang-Gung Institutional Animal Care and Use Committee.

### Statistical Analysis

The statistical analyses were conducted using t-tests, a one-way analysis of variance (ANOVA), and a Tukey’s honest significant difference post hoc test. The correlation analysis was conducted using spearman. The data are presented as the means ± standard deviations.

## Electronic supplementary material

Supplementary Information
