# Supplementary material for: Thyroid hormone inhibits growth of hepatoma cells through induction of miR-214
Source: Sci Rep. 2017 Nov 1;7:14868. doi: 10.1038/s41598-017-14864-1 (PMC5665905; doi:10.1038/s41598-017-14864-1)

Thyroid hormone inhibits growth of hepatoma cells through induction of miR-214

Po-Shuan Huang[1], Yang-Hsiang Lin[1], Hsiang-Cheng Chi[2], Pei-Yu Chen[1], Ya-Hui Huang[3], Chau-Ting Yeh[3], Chia-Siu Wang[4*] and Kwang-Huei Lin[1,3,5*]

[1]Department of Biochemistry, College of Medicine, Chang-Gung University, Taoyuan, Taiwan 333

[2]Radiation Biology Research Center, Institute for Radiological Research, Chang Gung University/Chang Gung Memorial Hospital, Linkou, Taoyuan, Taiwan

[3]Liver Research Center, Chang Gung Memorial Hospital, Linko, Taoyuan, Taiwan

[4]Department of General Surgery, Chang Gung Memorial Hospital, Chiayi 613, Taiwan

[5]Research Center for Chinese Herbal Medicine, College of Human Ecology, Chang Gung University of Science and Technology Taoyuan, Taiwan

Running title: miR-214 regulated by thyroid hormone

Keywords: microRNA-214; PIM-1; thyroid, receptor, hepatoma

*Equal contribution

To whom correspondence and reprint requests should be addressed: Dr. Kwang-Huei Lin, Department of Biochemistry, Chang-Gung University, E-mail: khlin@mail.cgu.edu.tw; or Dr. Chia-Siu Wang, Department of General Surgery,

Chang Gung Memorial Hospital, E-mail: wangcs@cgmh.org.tw

Supplementary Information

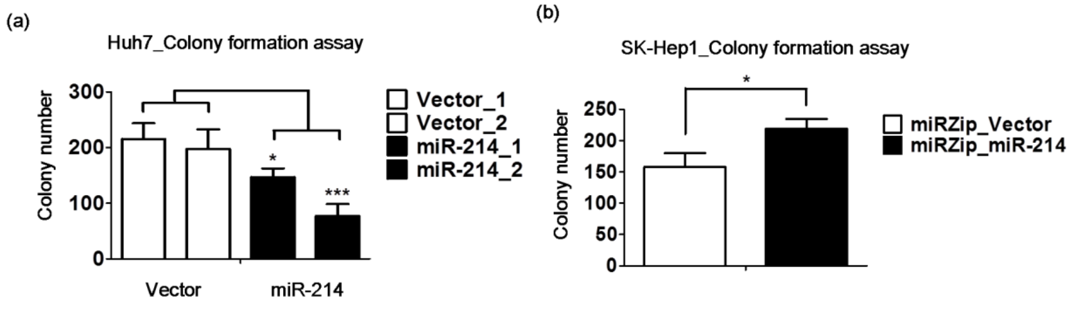

Fig.S1 miR-214 inhibits cell proliferation

Stable expression miR-214 Huh7 cell lines were established by transfect with miR-214 plasmid. (a) Huh7 hepatoma cell lines proliferation capacity was measured by Colony assay. Stable knockdown of miR-214 in SK-Hep-1 cell lines were established by infection the lentivirus of miRZip-miR-214 (Anti-miR-214). (b) SK-Hep-1 hepatoma cell lines proliferation capacity were measured by Colony assay.

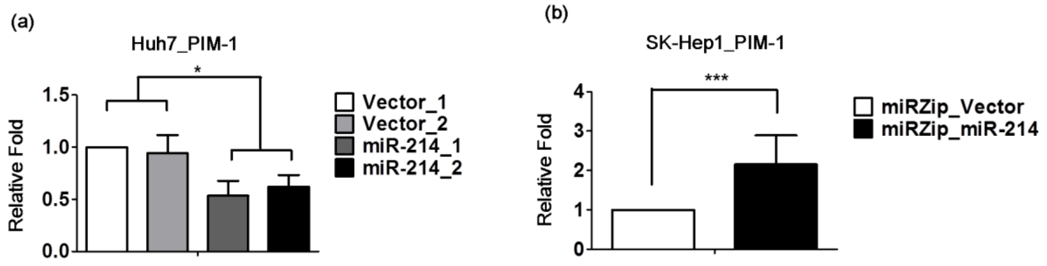

Fig.S2 PIM-1 is the target gene of miR-214

(a) PIM-1 protein levels in Huh7 stable expression and (b) SK-Hep-1 stable knockdown miR-214 cells measuring by western blot. β-actin was used as a loading control. Data are presented as means± s.d. of three independent experiments (*$p < 0.05$; **$p<0.005$; ***$p<0.001$ v.s. Vector).

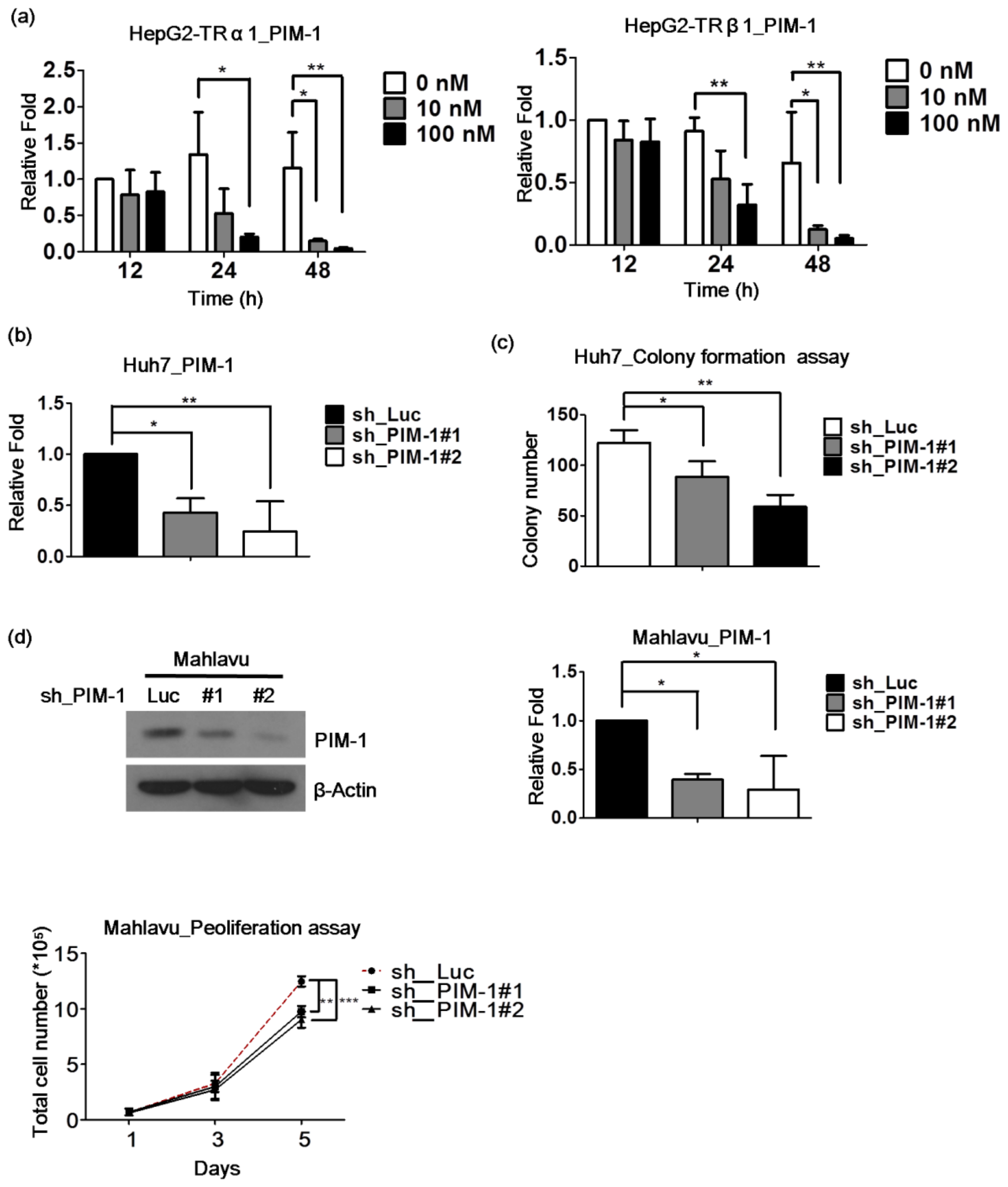

Fig.S3 PIM-1 is downregulated by T_3 and promotes cell proliferation

(a) HepG2-TR cell lines were treated with 0-100 nM T_3 for 12-48 hrs, and PIM-1 protein levels were measured by western blot. (b) Stable knockdown PIM-1 in Huh7 cell lines were established by transfect with shRNA PIM-1 plasmids. (c) Huh7 hepatoma cell lines proliferation capacity was measured by colony formation assay. (d) Stable knockdown PIM-1 in Mahlavu cell lines were established by transfect with shRNA PIM-1 plasmids. β-actin was used as a loading control. (e) Mahlavu hepatoma cell lines proliferation capacity was measured by proliferation assay. Data are

presented as means± s.d. of three independent experiments (*$p < 0.05$; **$p<0.005$ v.s. control).

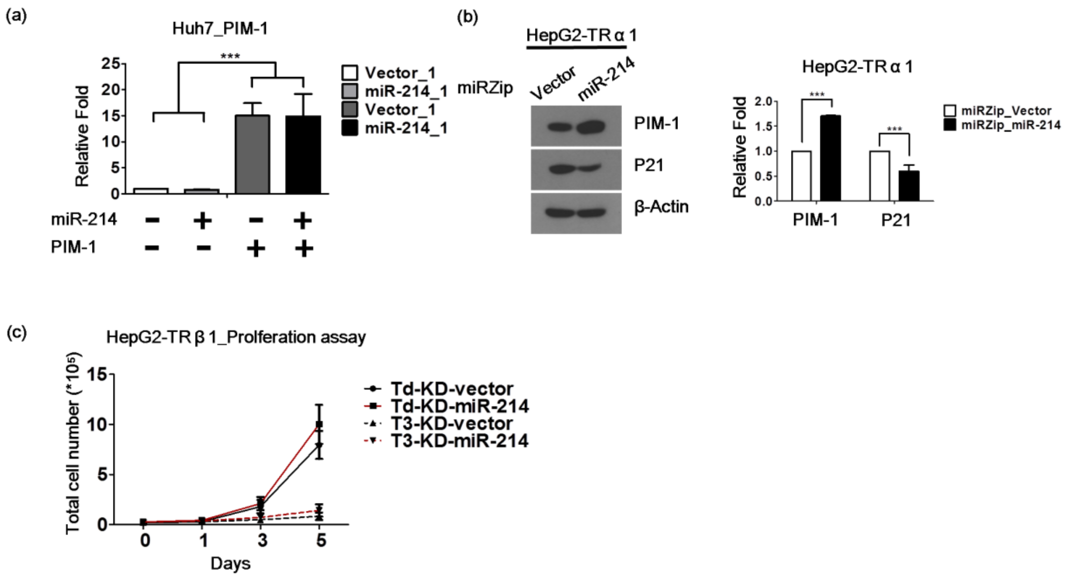

Fig.S4 T_3 inhibits cell proliferation through upregulation miR-214 via modulation of the PIM-1 pathway

(a) Re-expression of PIM-1 in miR-214 stable Huh7 cell lines, and PIM-1 protein levels were measuring by western blot. (b) PIM-1 and p21 protein expression were measuring in stable knockdown miR-214 HepG2-TRα1 cell lines by western blot. β-actin was used as a loading control. (c) HepG2-TRβ1 depletion of miR-214 cell lines were treated with 0-10 nM T_3, and proliferation capacity was measured. Data are presented as means± s.d. of three independent experiments (*$P<0.05$;**$P<0.01$ v.s. Vector).

Fig.1e

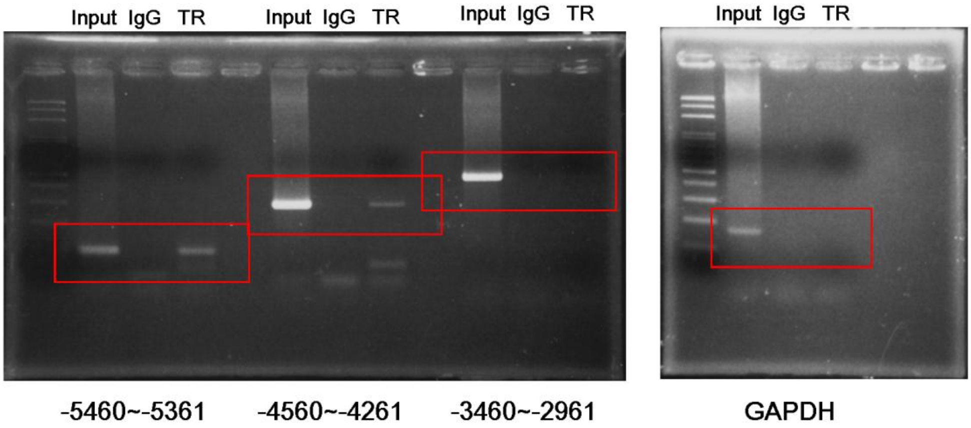

Input IgG TR
Input IgG TR
Input IgG TR
Input IgG TR
-5460~-5361
-4560~-4261
-3460~-2961
GAPDH

Fig.3 (b)

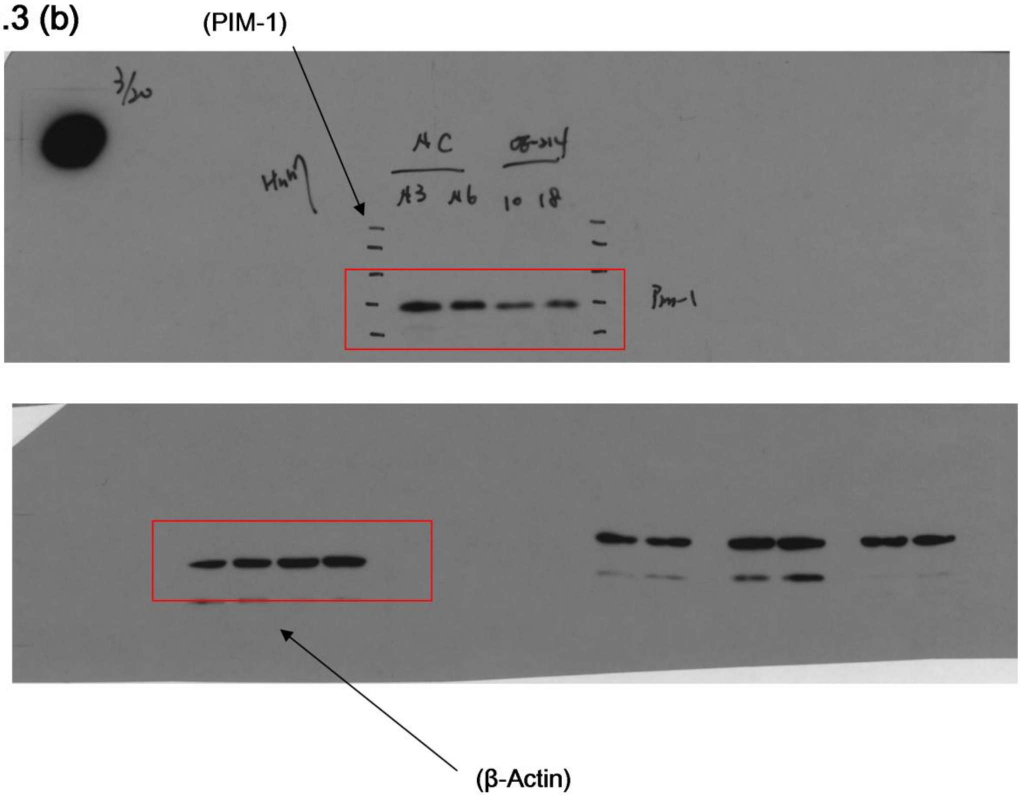

Fig.3 (c)

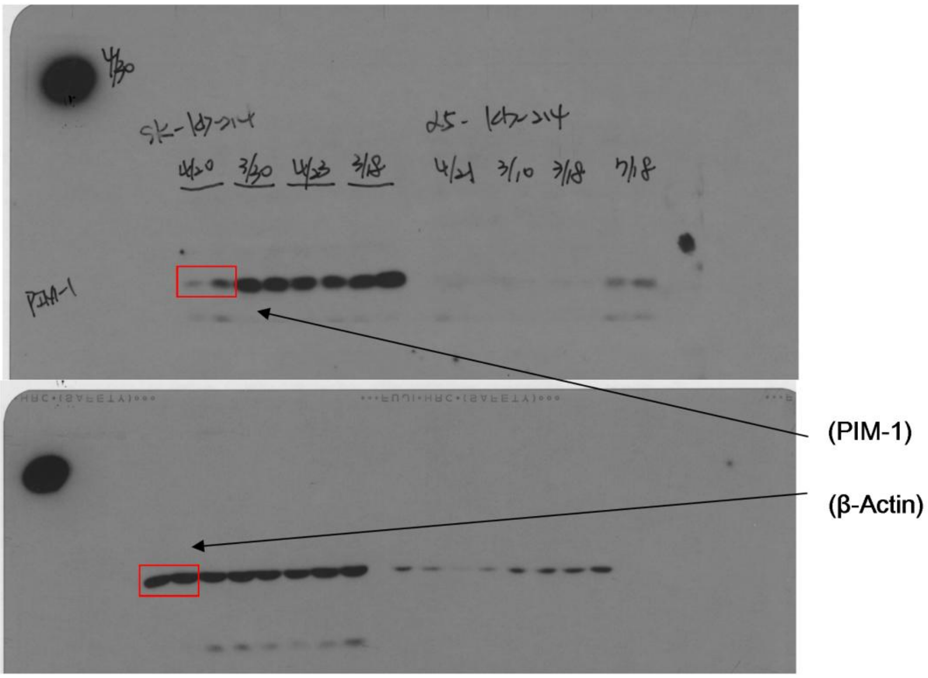

Fig.3 (d)

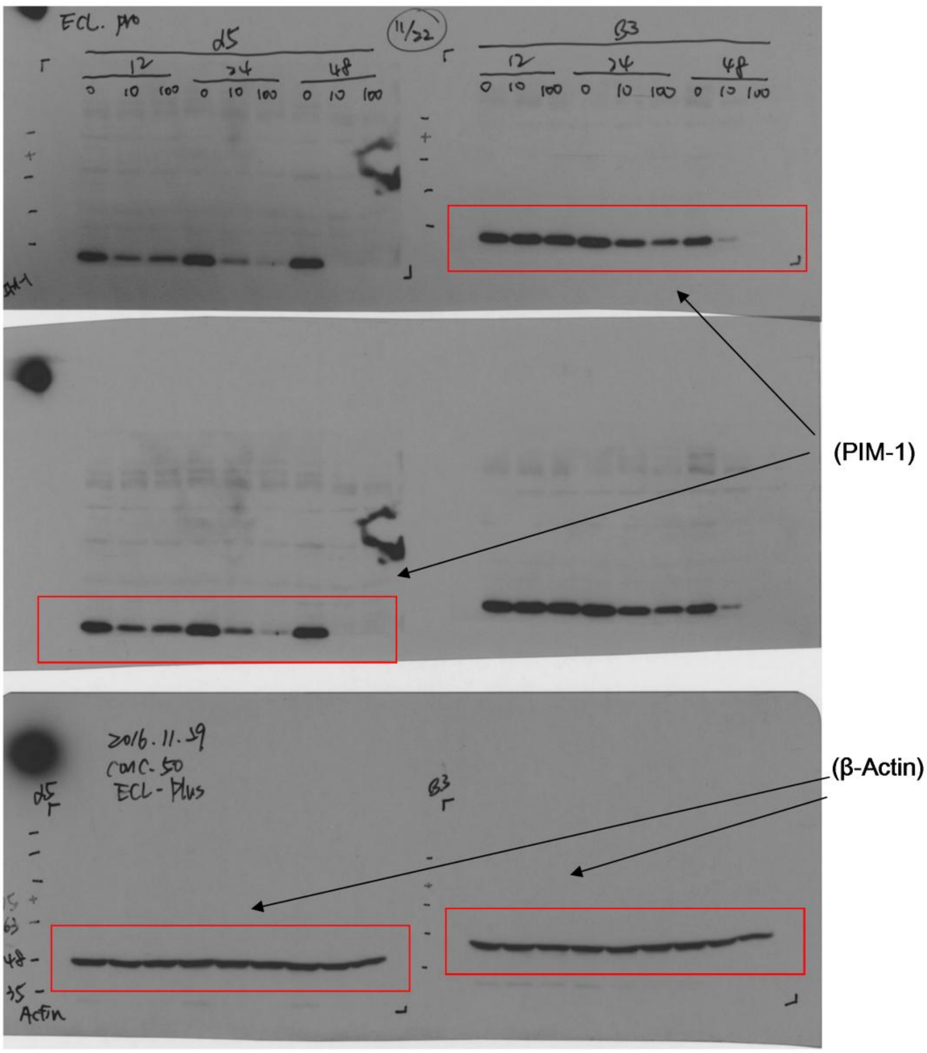

Fig.3 (e)

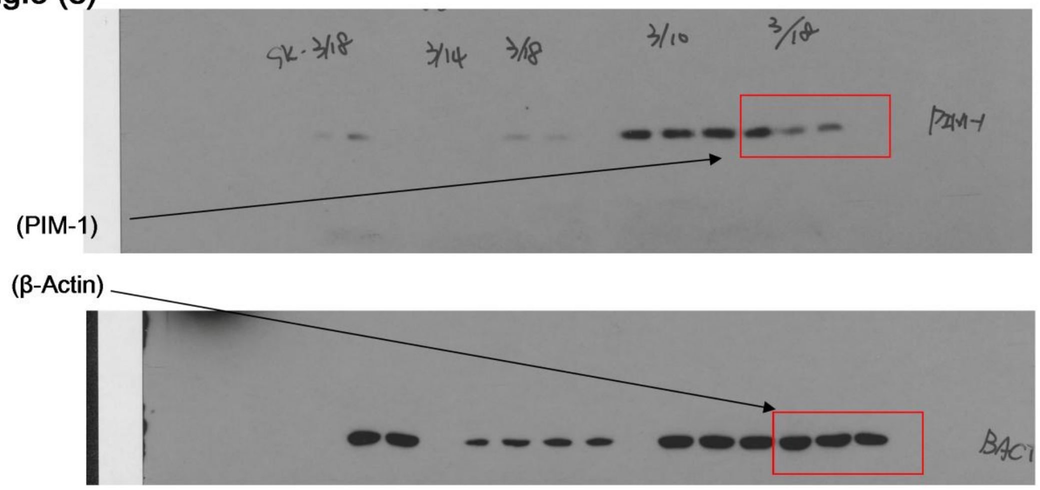

Fig.4 (a)

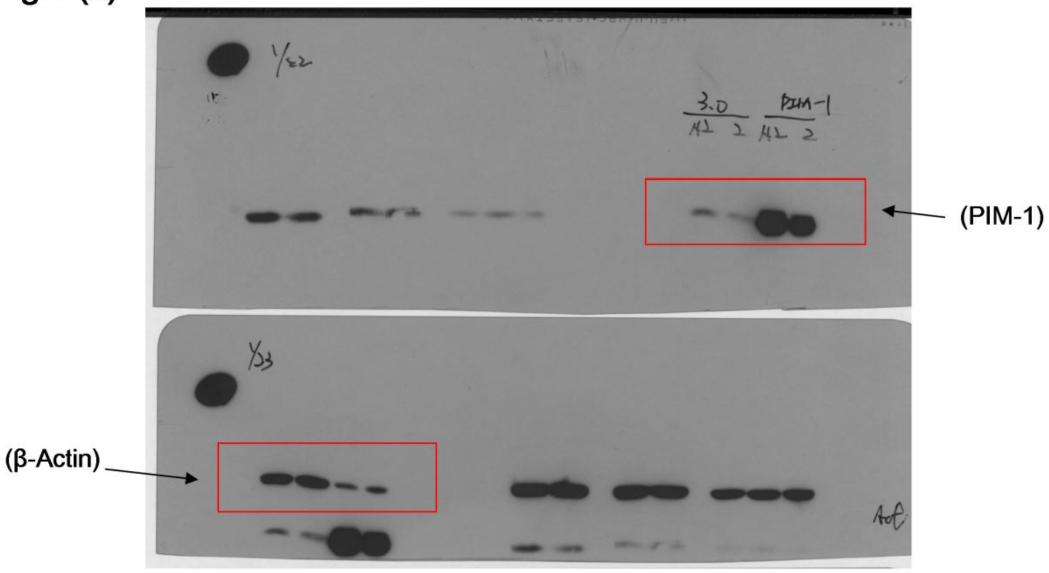

Fig.4 (c)

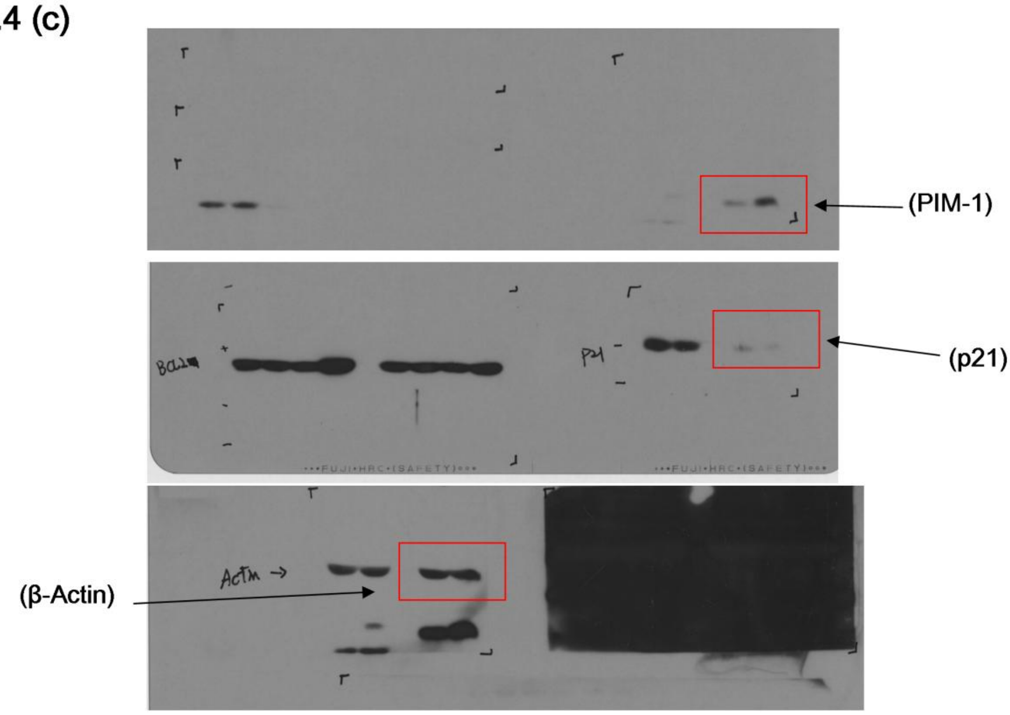

Fig.4 (d)

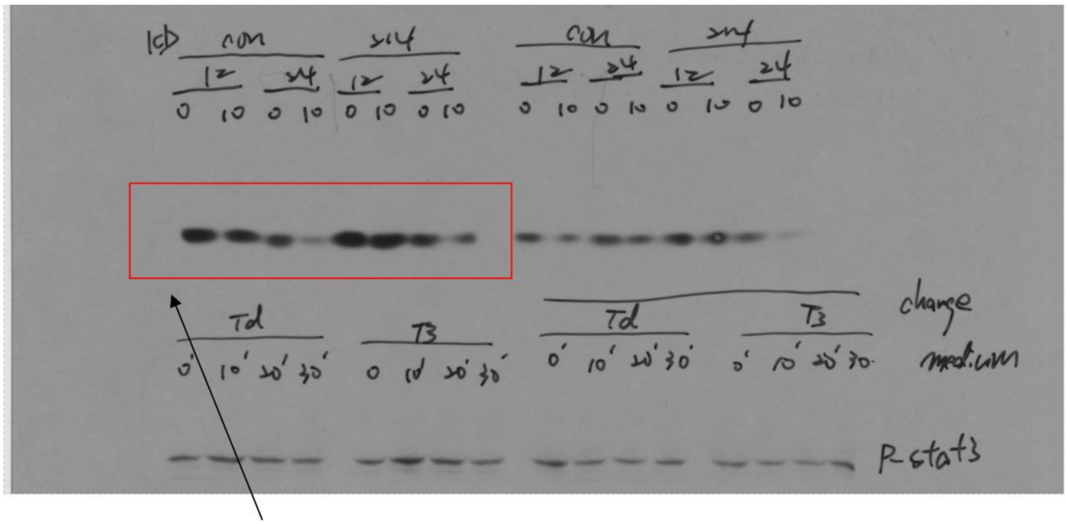

(PIM-1)

(β-Actin)

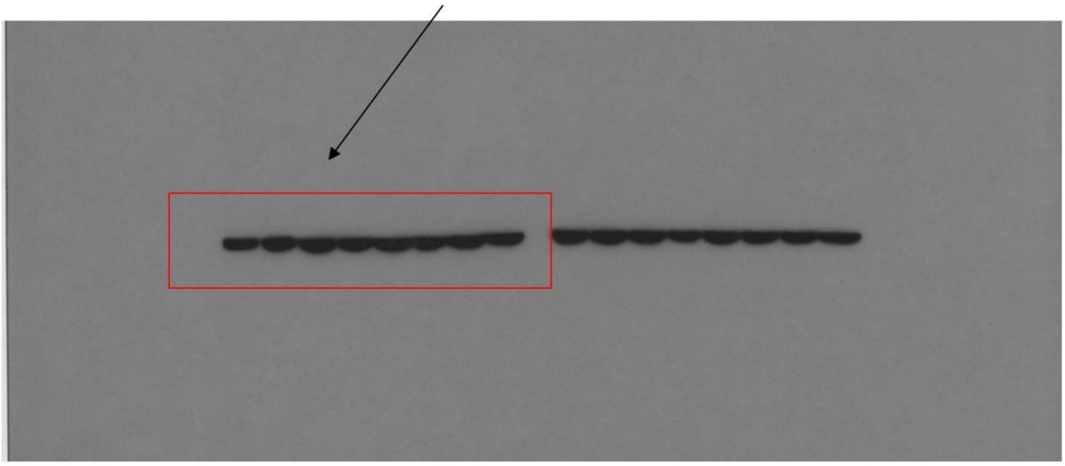

Fig.5 (b)

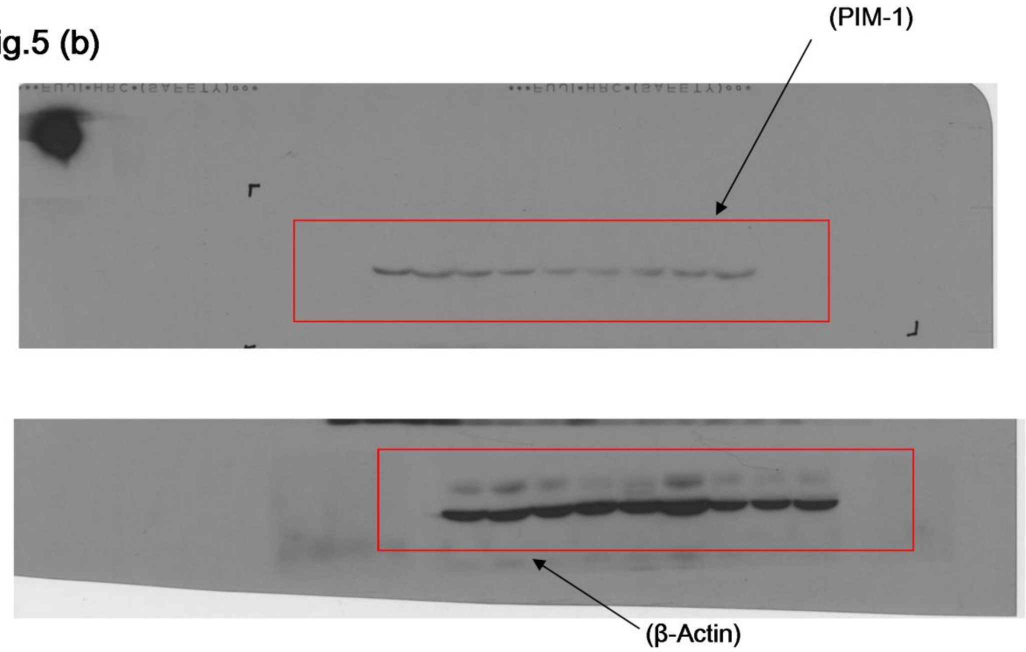

Fig.5 (c)

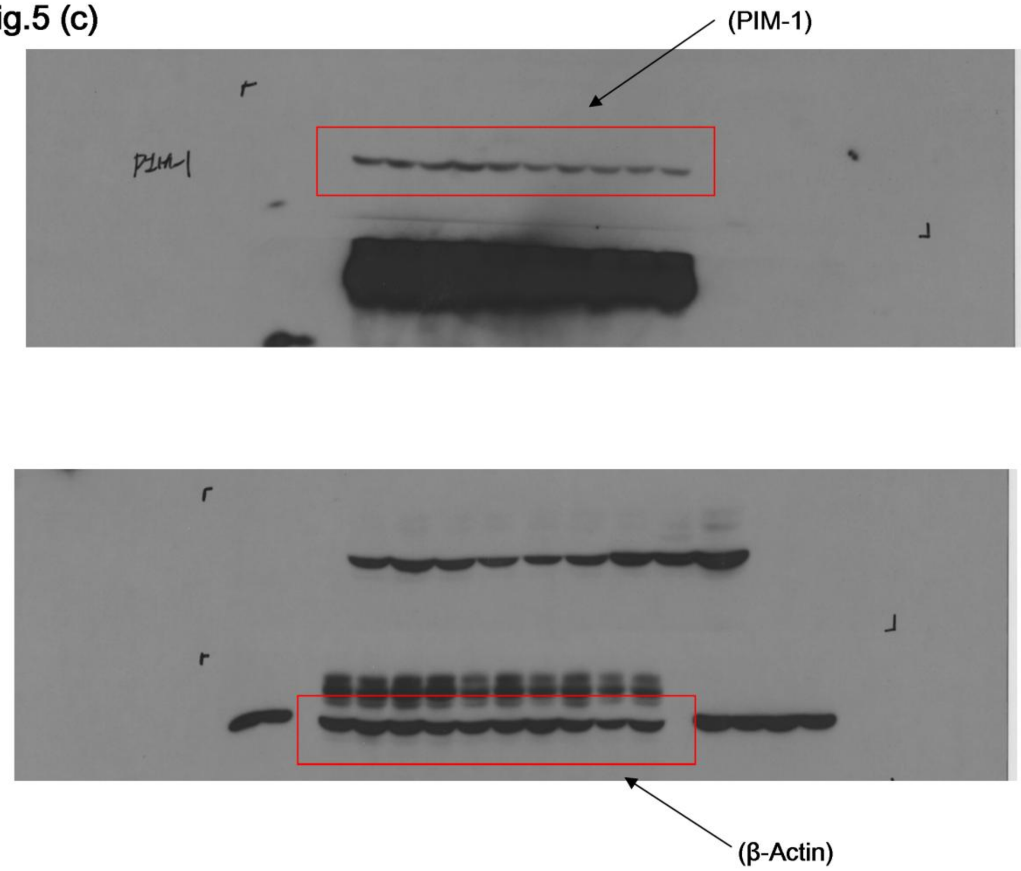

Fig.5 (g)

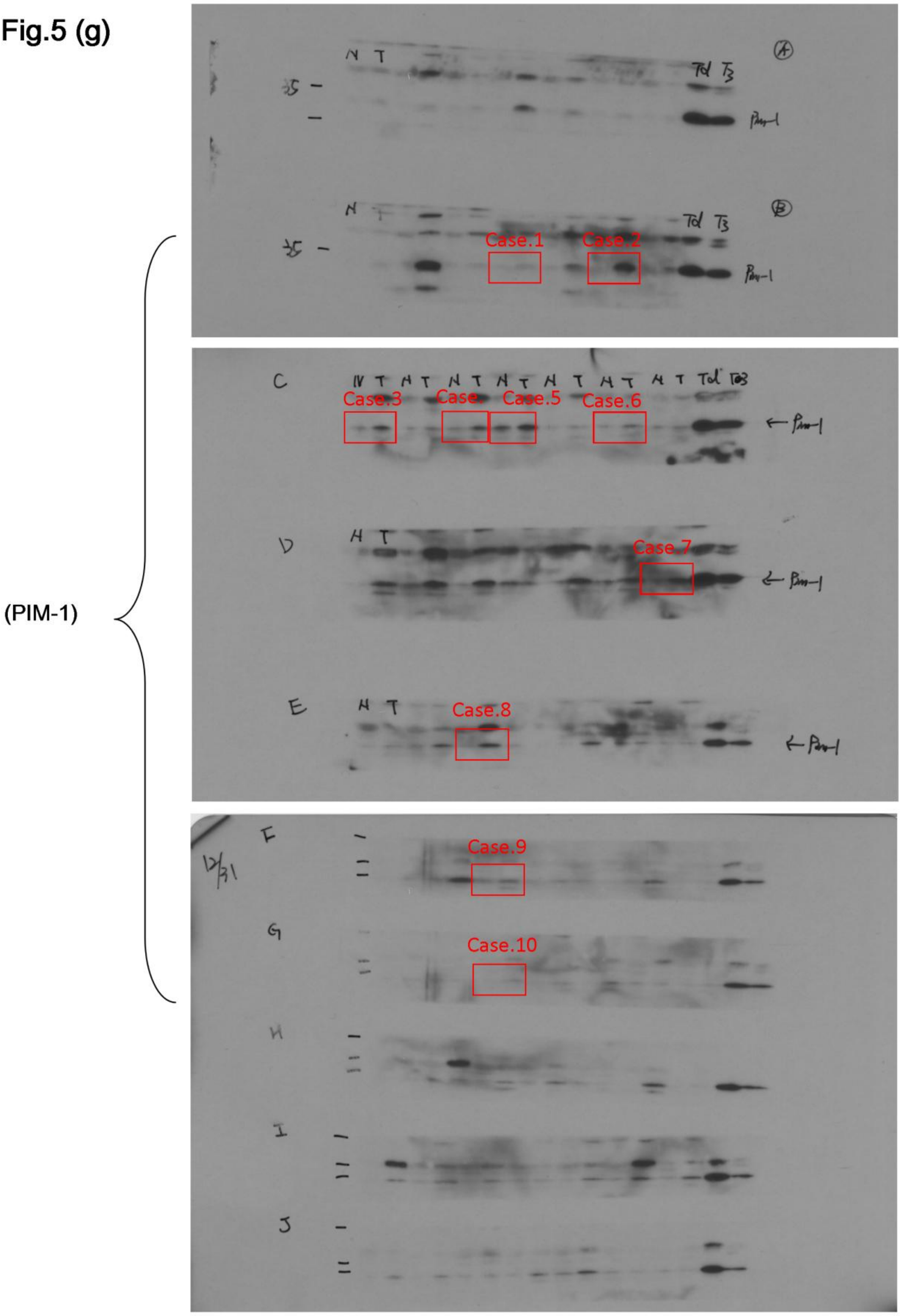
(PIM-1)
Case.1
Case.2
Case.3
Case.
Case.5
Case.6
Case.7
Case.8
Case.9
Case.10

Fig.5 (g)

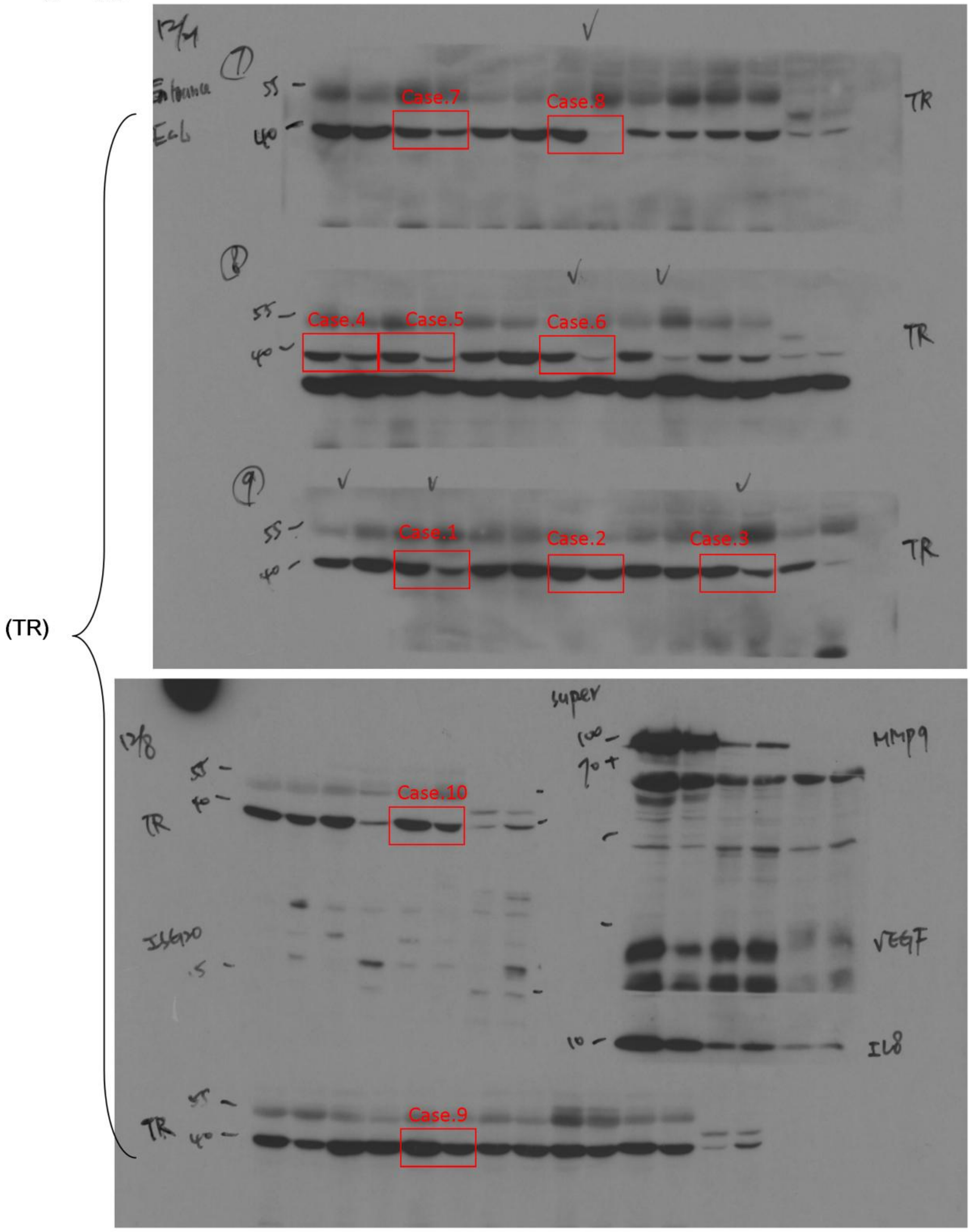
(TR)
Case.7
Case.8
Case.4
Case.5
Case.6
Case.1
Case.2
Case.3
Case.10
Case.9
TR
MMP9
VEGF
IL8
super

Fig.5 (g)

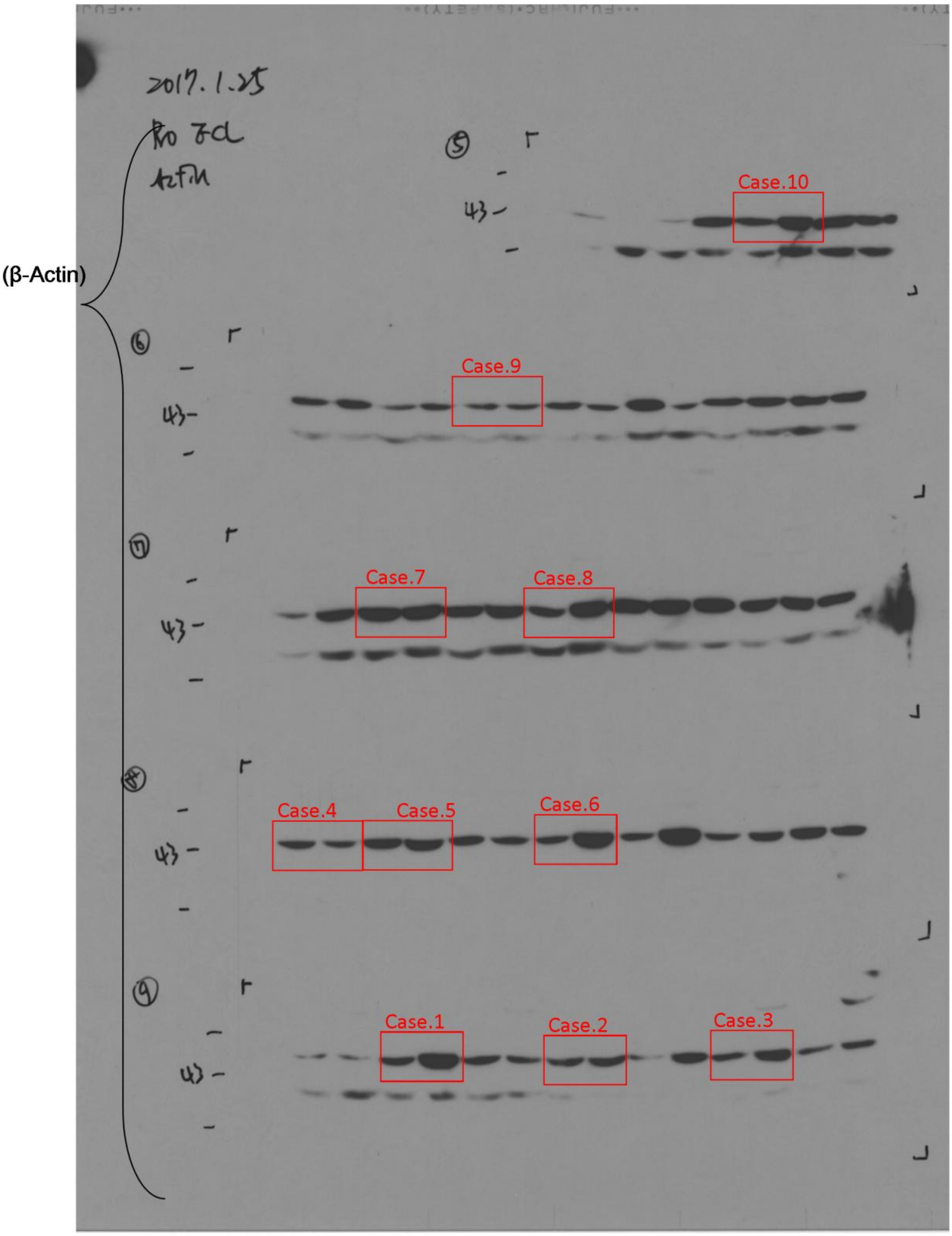
(β-Actin)
2017.1.25
Case.10
43
Case.9
43
Case.7
Case.8
43
Case.4
Case.5
Case.6
43
Case.1
Case.2
Case.3
43

Fig.S3

(**d**) (PIM-1, β-Actin)

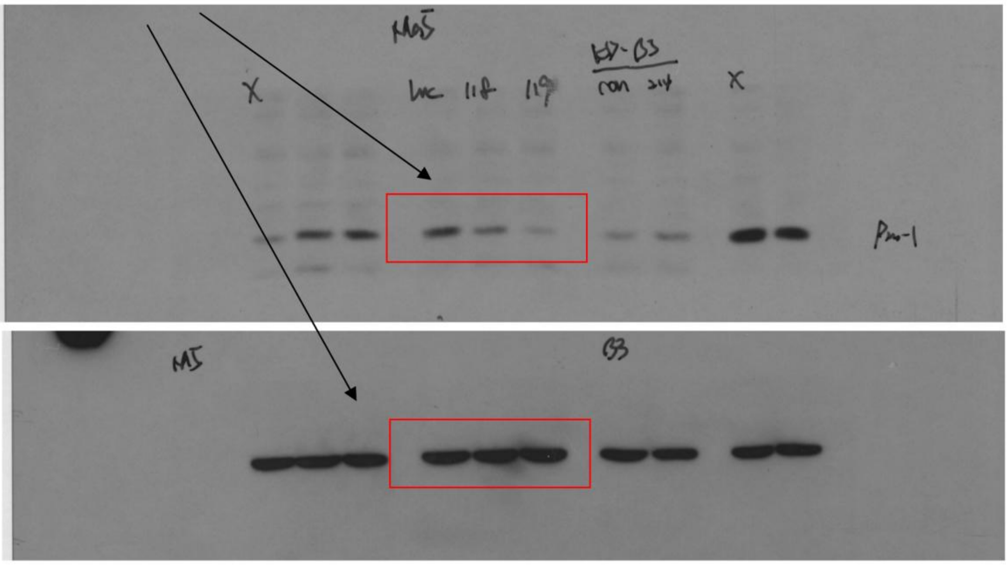

Fig.S3

(**b**)

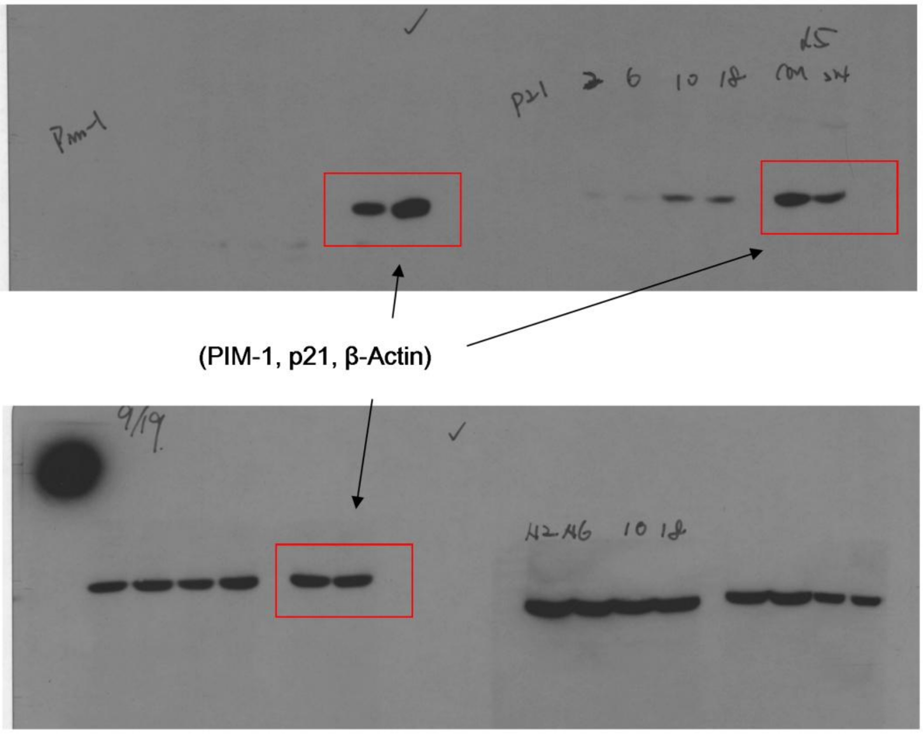

Supplement: Supplementary file 1 — Supplementary Information [file 41598_2017_14864_MOESM1_ESM.pdf]
